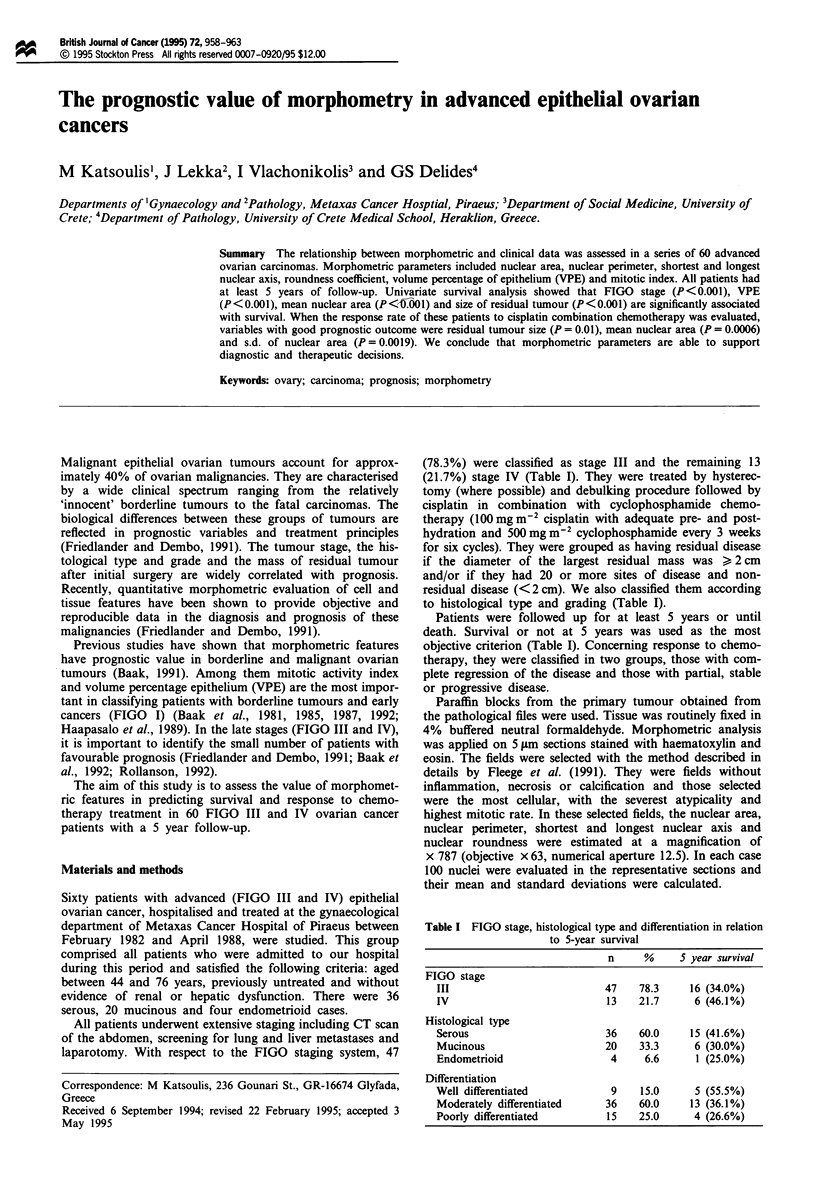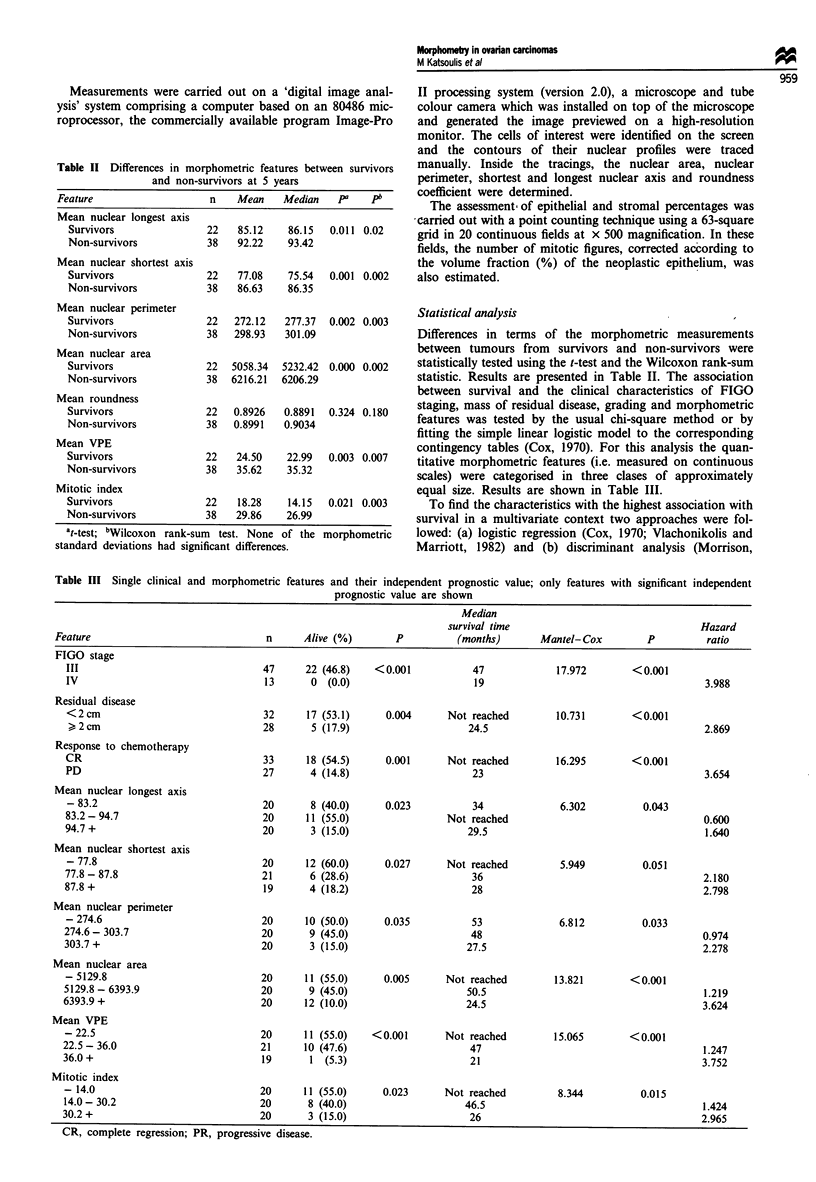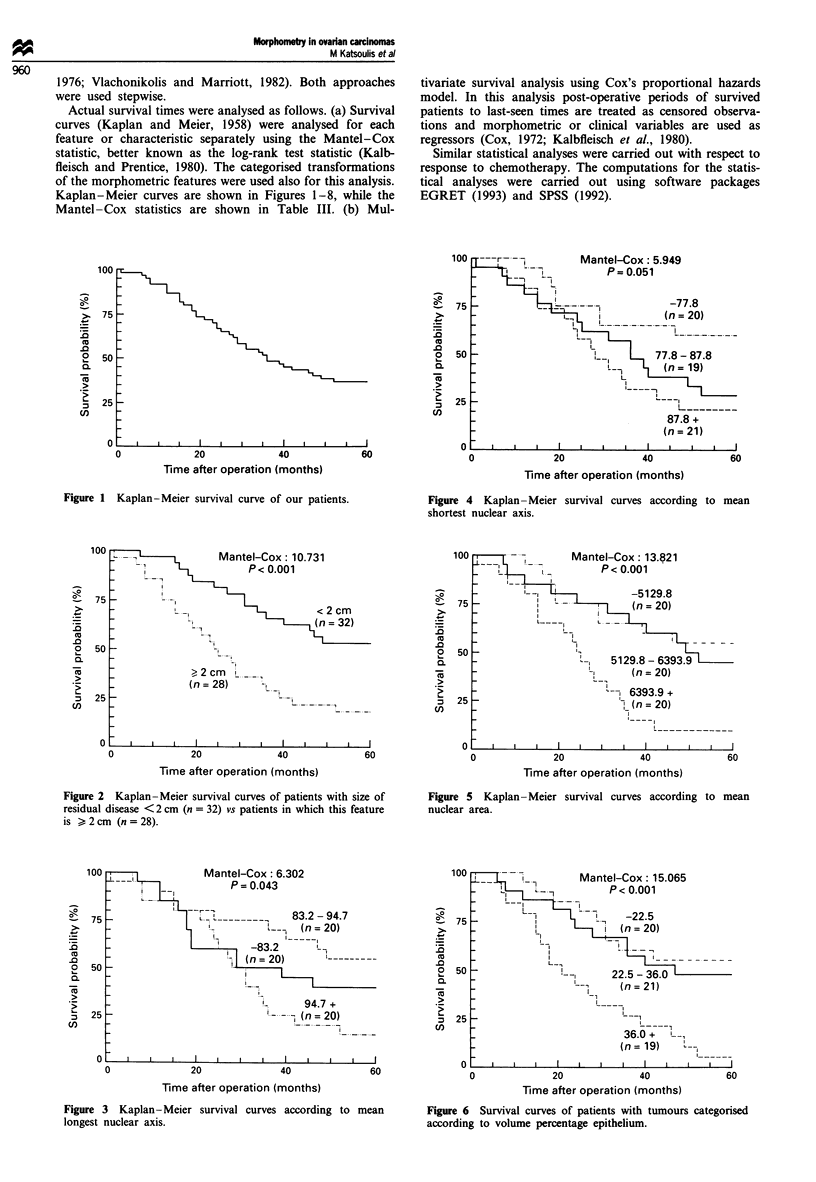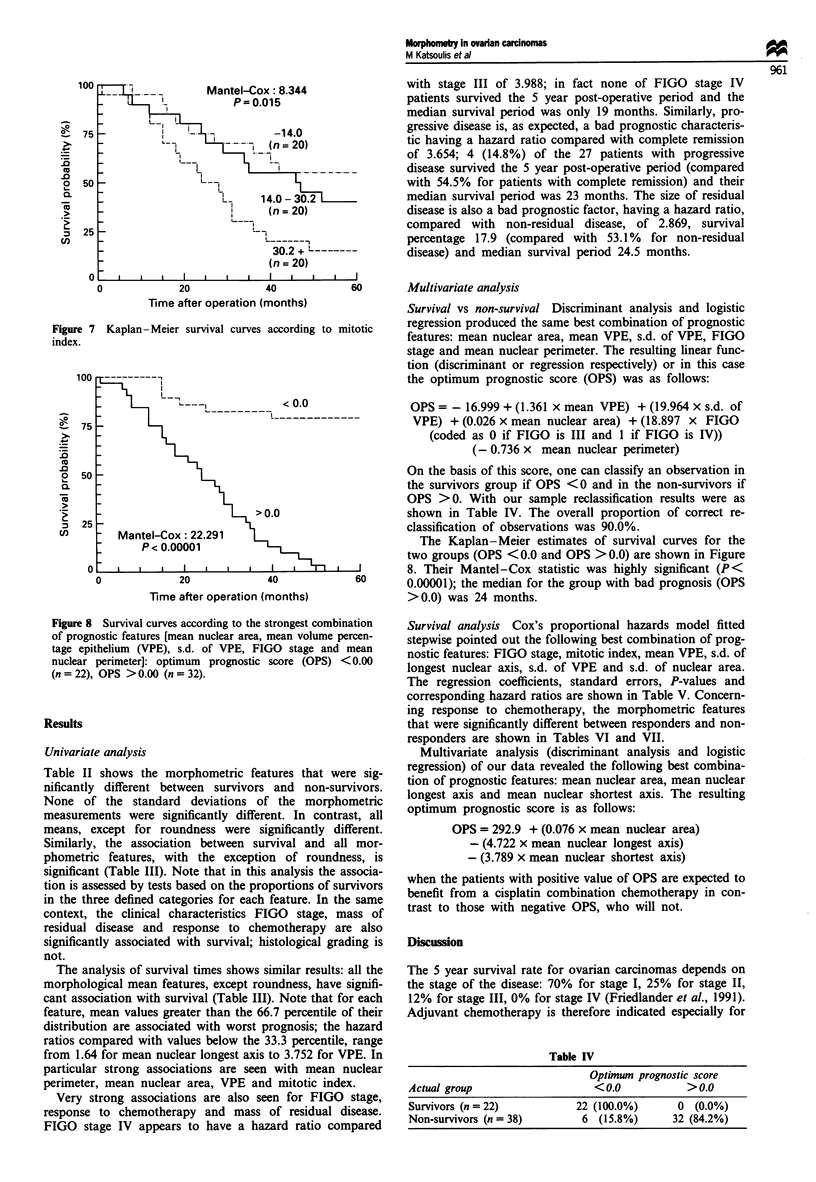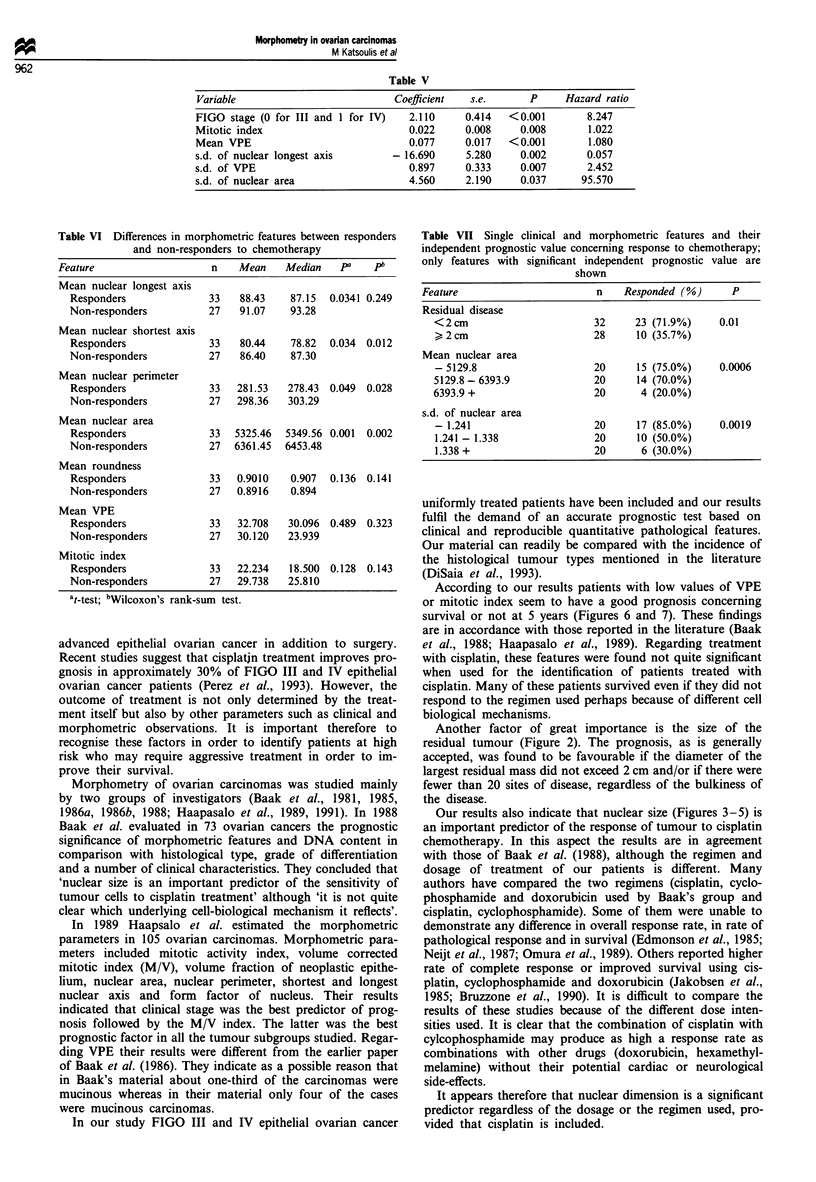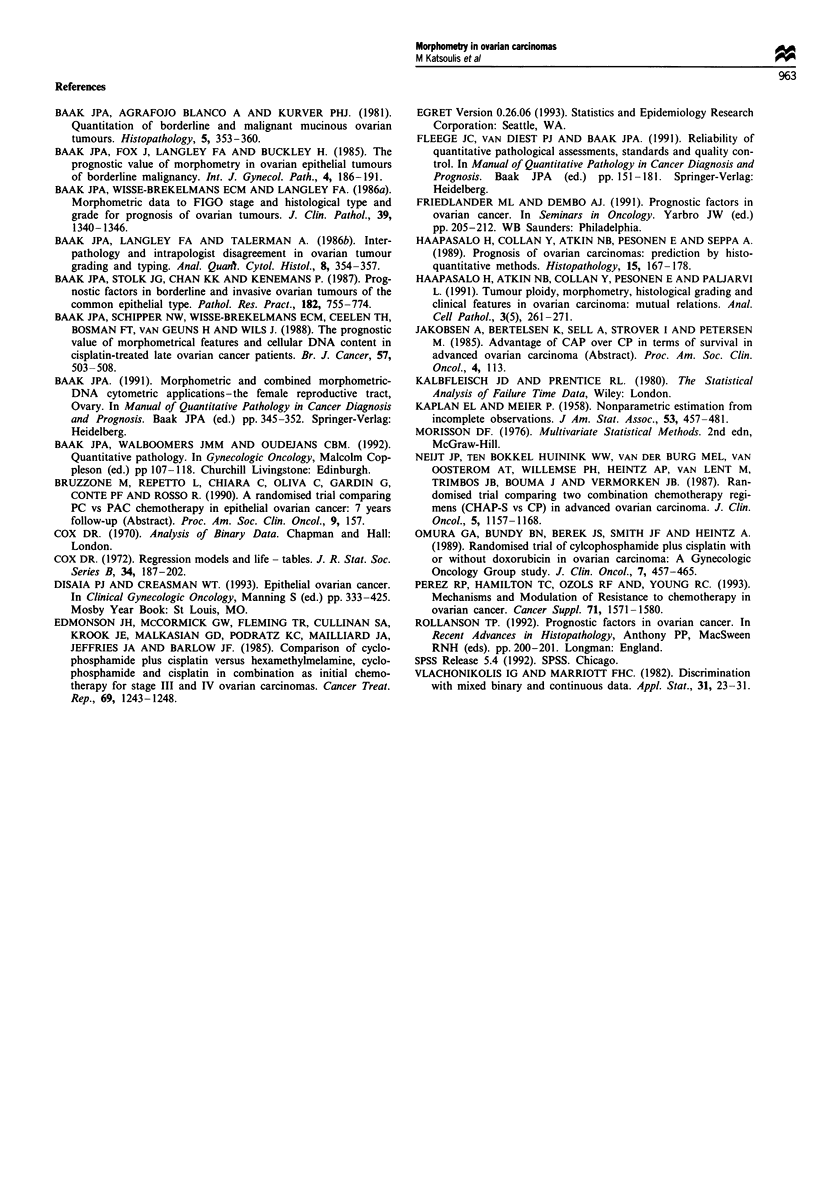# The prognostic value of morphometry in advanced epithelial ovarian cancers.

**DOI:** 10.1038/bjc.1995.441

**Published:** 1995-10

**Authors:** M. Katsoulis, J. Lekka, I. Vlachonikolis, G. S. Delides

**Affiliations:** Department of Gynaecology, Metaxas Cancer Hospital, Piraeus, Greece.

## Abstract

The relationship between morphometric and clinical data was assessed in a series of 60 advanced ovarian carcinomas. Morphometric parameters included nuclear area, nuclear perimeter, shortest and longest nuclear axis, roundness coefficient, volume percentage of epithelium (VPE) and mitotic index. All patients had at least 5 years of follow-up. Univariate survival analysis showed that FIGO stage (P < 0.001), VPE (P < 0.001), mean nuclear area (P < 0.001) and size of residual tumour (P < 0.001) are significantly associated with survival. When the response rate of these patients to cisplatin combination chemotherapy was evaluated, variables with good prognostic outcome were residual tumour size (P = 0.01), mean nuclear area (P = 0.0006) and s.d. of nuclear area (P = 0.0019). We conclude that morphometric parameters are able to support diagnostic and therapeutic decisions.


British Journal of Cancer (1995) 72, 958–963
© 1995 Stockton Press All rights reserved 0007-0920/95 $12.00

### The prognostic value of morphometry in advanced epithelial ovarian cancers

M Katsoulis[1], J Lekka[2], I Vlachonikolis[3] and GS Delides[4]

*Departments of [1]Gynaecology and [2]Pathology, Metaxas Cancer Hosptial, Piraeus; [3]Department of Social Medicine, University of Crete; [4]Department of Pathology, University of Crete Medical School, Heraklion, Greece.*

**Summary** The relationship between morphometric and clinical data was assessed in a series of 60 advanced ovarian carcinomas. Morphometric parameters included nuclear area, nuclear perimeter, shortest and longest nuclear axis, roundness coefficient, volume percentage of epithelium (VPE) and mitotic index. All patients had at least 5 years of follow-up. Univariate survival analysis showed that FIGO stage ($P<0.001$), VPE ($P<0.001$), mean nuclear area ($P<0.001$) and size of residual tumour ($P<0.001$) are significantly associated with survival. When the response rate of these patients to cisplatin combination chemotherapy was evaluated, variables with good prognostic outcome were residual tumour size ($P=0.01$), mean nuclear area ($P=0.0006$) and s.d. of nuclear area ($P=0.0019$). We conclude that morphometric parameters are able to support diagnostic and therapeutic decisions.

**Keywords:** ovary; carcinoma; prognosis; morphometry

Malignant epithelial ovarian tumours account for approximately 40% of ovarian malignancies. They are characterised by a wide clinical spectrum ranging from the relatively 'innocent' borderline tumours to the fatal carcinomas. The biological differences between these groups of tumours are reflected in prognostic variables and treatment principles (Friedlander and Dembo, 1991). The tumour stage, the histological type and grade and the mass of residual tumour after initial surgery are widely correlated with prognosis. Recently, quantitative morphometric evaluation of cell and tissue features have been shown to provide objective and reproducible data in the diagnosis and prognosis of these malignancies (Friedlander and Dembo, 1991).

Previous studies have shown that morphometric features have prognostic value in borderline and malignant ovarian tumours (Baak, 1991). Among them mitotic activity index and volume percentage epithelium (VPE) are the most important in classifying patients with borderline tumours and early cancers (FIGO I) (Baak *et al.*, 1981, 1985, 1987, 1992; Haapasalo *et al.*, 1989). In the late stages (FIGO III and IV), it is important to identify the small number of patients with favourable prognosis (Friedlander and Dembo, 1991; Baak *et al.*, 1992; Rollanson, 1992).

The aim of this study is to assess the value of morphometric features in predicting survival and response to chemotherapy treatment in 60 FIGO III and IV ovarian cancer patients with a 5 year follow-up.

### Materials and methods

Sixty patients with advanced (FIGO III and IV) epithelial ovarian cancer, hospitalised and treated at the gynaecological department of Metaxas Cancer Hospital of Piraeus between February 1982 and April 1988, were studied. This group comprised all patients who were admitted to our hospital during this period and satisfied the following criteria: aged between 44 and 76 years, previously untreated and without evidence of renal or hepatic dysfunction. There were 36 serous, 20 mucinous and four endometrioid cases.

All patients underwent extensive staging including CT scan of the abdomen, screening for lung and liver metastases and laparotomy. With respect to the FIGO staging system, 47 (78.3%) were classified as stage III and the remaining 13 (21.7%) stage IV (Table I). They were treated by hysterectomy (where possible) and debulking procedure followed by cisplatin in combination with cyclophosphamide chemotherapy ($100\ \mathrm{mg\ m^{-2}}$ cisplatin with adequate pre- and post-hydration and $500\ \mathrm{mg\ m^{-2}}$ cyclophosphamide every 3 weeks for six cycles). They were grouped as having residual disease if the diameter of the largest residual mass was $\geqslant 2$ cm and/or if they had 20 or more sites of disease and non-residual disease ($<2$ cm). We also classified them according to histological type and grading (Table I).

Patients were followed up for at least 5 years or until death. Survival or not at 5 years was used as the most objective criterion (Table I). Concerning response to chemotherapy, they were classified in two groups, those with complete regression of the disease and those with partial, stable or progressive disease.

Paraffin blocks from the primary tumour obtained from the pathological files were used. Tissue was routinely fixed in 4% buffered neutral formaldehyde. Morphometric analysis was applied on 5 μm sections stained with haematoxylin and eosin. The fields were selected with the method described in details by Fleege *et al.* (1991). They were fields without inflammation, necrosis or calcification and those selected were the most cellular, with the severest atypicality and highest mitotic rate. In these selected fields, the nuclear area, nuclear perimeter, shortest and longest nuclear axis and nuclear roundness were estimated at a magnification of × 787 (objective × 63, numerical aperture 12.5). In each case 100 nuclei were evaluated in the representative sections and their mean and standard deviations were calculated.

**Table I** FIGO stage, histological type and differentiation in relation to 5-year survival

| | n | % | *5 year survival* |
|---|---|---|---|
| FIGO stage | | | |
| III | 47 | 78.3 | 16 (34.0%) |
| IV | 13 | 21.7 | 6 (46.1%) |
| Histological type | | | |
| Serous | 36 | 60.0 | 15 (41.6%) |
| Mucinous | 20 | 33.3 | 6 (30.0%) |
| Endometrioid | 4 | 6.6 | 1 (25.0%) |
| Differentiation | | | |
| Well differentiated | 9 | 15.0 | 5 (55.5%) |
| Moderately differentiated | 36 | 60.0 | 13 (36.1%) |
| Poorly differentiated | 15 | 25.0 | 4 (26.6%) |

Correspondence: M Katsoulis, 236 Gounari St., GR-16674 Glyfada, Greece
Received 6 September 1994; revised 22 February 1995; accepted 3 May 1995

Measurements were carried out on a 'digital image analysis' system comprising a computer based on an 80486 microprocessor, the commercially available program Image-Pro II processing system (version 2.0), a microscope and tube colour camera which was installed on top of the microscope and generated the image previewed on a high-resolution monitor. The cells of interest were identified on the screen and the contours of their nuclear profiles were traced manually. Inside the tracings, the nuclear area, nuclear perimeter, shortest and longest nuclear axis and roundness coefficient were determined.

The assessment of epithelial and stromal percentages was carried out with a point counting technique using a 63-square grid in 20 continuous fields at × 500 magnification. In these fields, the number of mitotic figures, corrected according to the volume fraction (%) of the neoplastic epithelium, was also estimated.

**Table II** Differences in morphometric features between survivors and non-survivors at 5 years

| *Feature* | n | *Mean* | *Median* | $P^a$ | $P^b$ |
|---|---|---|---|---|---|
| Mean nuclear longest axis | | | | | |
| Survivors | 22 | 85.12 | 86.15 | 0.011 | 0.02 |
| Non-survivors | 38 | 92.22 | 93.42 | | |
| Mean nuclear shortest axis | | | | | |
| Survivors | 22 | 77.08 | 75.54 | 0.001 | 0.002 |
| Non-survivors | 38 | 86.63 | 86.35 | | |
| Mean nuclear perimeter | | | | | |
| Survivors | 22 | 272.12 | 277.37 | 0.002 | 0.003 |
| Non-survivors | 38 | 298.93 | 301.09 | | |
| Mean nuclear area | | | | | |
| Survivors | 22 | 5058.34 | 5232.42 | 0.000 | 0.002 |
| Non-survivors | 38 | 6216.21 | 6206.29 | | |
| Mean roundness | | | | | |
| Survivors | 22 | 0.8926 | 0.8891 | 0.324 | 0.180 |
| Non-survivors | 38 | 0.8991 | 0.9034 | | |
| Mean VPE | | | | | |
| Survivors | 22 | 24.50 | 22.99 | 0.003 | 0.007 |
| Non-survivors | 38 | 35.62 | 35.32 | | |
| Mitotic index | | | | | |
| Survivors | 22 | 18.28 | 14.15 | 0.021 | 0.003 |
| Non-survivors | 38 | 29.86 | 26.99 | | |

[a]*t*-test; [b]Wilcoxon rank-sum test. None of the morphometric standard deviations had significant differences.

*Statistical analysis*

Differences in terms of the morphometric measurements between tumours from survivors and non-survivors were statistically tested using the *t*-test and the Wilcoxon rank-sum statistic. Results are presented in Table II. The association between survival and the clinical characteristics of FIGO staging, mass of residual disease, grading and morphometric features was tested by the usual chi-square method or by fitting the simple linear logistic model to the corresponding contingency tables (Cox, 1970). For this analysis the quantitative morphometric features (i.e. measured on continuous scales) were categorised in three clases of approximately equal size. Results are shown in Table III.

To find the characteristics with the highest association with survival in a multivariate context two approaches were followed: (a) logistic regression (Cox, 1970; Vlachonikolis and Marriott, 1982) and (b) discriminant analysis (Morrison,

**Table III** Single clinical and morphometric features and their independent prognostic value; only features with significant independent prognostic value are shown

| *Feature* | n | *Alive (%)* | *P* | *Median survival time (months)* | *Mantel–Cox* | *P* | *Hazard ratio* |
|---|---|---|---|---|---|---|---|
| FIGO stage | | | | | | | |
| III | 47 | 22 (46.8) | <0.001 | 47 | 17.972 | <0.001 | |
| IV | 13 | 0 (0.0) | | 19 | | | 3.988 |
| Residual disease | | | | | | | |
| <2 cm | 32 | 17 (53.1) | 0.004 | Not reached | 10.731 | <0.001 | |
| ⩾2 cm | 28 | 5 (17.9) | | 24.5 | | | 2.869 |
| Response to chemotherapy | | | | | | | |
| CR | 33 | 18 (54.5) | 0.001 | Not reached | 16.295 | <0.001 | |
| PD | 27 | 4 (14.8) | | 23 | | | 3.654 |
| Mean nuclear longest axis | | | | | | | |
| – 83.2 | 20 | 8 (40.0) | 0.023 | 34 | 6.302 | 0.043 | |
| 83.2 – 94.7 | 20 | 11 (55.0) | | Not reached | | | 0.600 |
| 94.7 + | 20 | 3 (15.0) | | 29.5 | | | 1.640 |
| Mean nuclear shortest axis | | | | | | | |
| – 77.8 | 20 | 12 (60.0) | 0.027 | Not reached | 5.949 | 0.051 | |
| 77.8 – 87.8 | 21 | 6 (28.6) | | 36 | | | 2.180 |
| 87.8 + | 19 | 4 (18.2) | | 28 | | | 2.798 |
| Mean nuclear perimeter | | | | | | | |
| – 274.6 | 20 | 10 (50.0) | 0.035 | 53 | 6.812 | 0.033 | |
| 274.6 – 303.7 | 20 | 9 (45.0) | | 48 | | | 0.974 |
| 303.7 + | 20 | 3 (15.0) | | 27.5 | | | 2.278 |
| Mean nuclear area | | | | | | | |
| – 5129.8 | 20 | 11 (55.0) | 0.005 | Not reached | 13.821 | <0.001 | |
| 5129.8 – 6393.9 | 20 | 9 (45.0) | | 50.5 | | | 1.219 |
| 6393.9 + | 20 | 12 (10.0) | | 24.5 | | | 3.624 |
| Mean VPE | | | | | | | |
| – 22.5 | 20 | 11 (55.0) | <0.001 | Not reached | 15.065 | <0.001 | |
| 22.5 – 36.0 | 21 | 10 (47.6) | | 47 | | | 1.247 |
| 36.0 + | 19 | 1 (5.3) | | 21 | | | 3.752 |
| Mitotic index | | | | | | | |
| – 14.0 | 20 | 11 (55.0) | 0.023 | Not reached | 8.344 | 0.015 | |
| 14.0 – 30.2 | 20 | 8 (40.0) | | 46.5 | | | 1.424 |
| 30.2 + | 20 | 3 (15.0) | | 26 | | | 2.965 |

CR, complete regression; PR, progressive disease.

1976; Vlachonikolis and Marriott, 1982). Both approaches were used stepwise.

Actual survival times were analysed as follows. (a) Survival curves (Kaplan and Meier, 1958) were analysed for each feature or characteristic separately using the Mantel–Cox statistic, better known as the log-rank test statistic (Kalbfleisch and Prentice, 1980). The categorised transformations of the morphometric features were used also for this analysis. Kaplan–Meier curves are shown in Figures 1–8, while the Mantel–Cox statistics are shown in Table III. (b) Multivariate survival analysis using Cox's proportional hazards model. In this analysis post-operative periods of survived patients to last-seen times are treated as censored observations and morphometric or clinical variables are used as regressors (Cox, 1972; Kalbfleisch et al., 1980).

Similar statistical analyses were carried out with respect to response to chemotherapy. The computations for the statistical analyses were carried out using software packages EGRET (1993) and SPSS (1992).

**Figure 1** Kaplan–Meier survival curve of our patients.

**Figure 2** Kaplan–Meier survival curves of patients with size of residual disease $<2$ cm ($n = 32$) vs patients in which this feature is $\geqslant 2$ cm ($n = 28$).

**Figure 3** Kaplan–Meier survival curves according to mean longest nuclear axis.

**Figure 4** Kaplan–Meier survival curves according to mean shortest nuclear axis.

**Figure 5** Kaplan–Meier survival curves according to mean nuclear area.

**Figure 6** Survival curves of patients with tumours categorised according to volume percentage epithelium.

**Figure 7** Kaplan–Meier survival curves according to mitotic index.

**Figure 8** Survival curves according to the strongest combination of prognostic features [mean nuclear area, mean volume percentage epithelium (VPE), s.d. of VPE, FIGO stage and mean nuclear perimeter]: optimum prognostic score (OPS) $<0.00$ ($n = 22$), OPS $>0.00$ ($n = 32$).

### Results

### *Univariate analysis*

Table II shows the morphometric features that were significantly different between survivors and non-survivors. None of the standard deviations of the morphometric measurements were significantly different. In contrast, all means, except for roundness were significantly different. Similarly, the association between survival and all morphometric features, with the exception of roundness, is significant (Table III). Note that in this analysis the association is assessed by tests based on the proportions of survivors in the three defined categories for each feature. In the same context, the clinical characteristics FIGO stage, mass of residual disease and response to chemotherapy are also significantly associated with survival; histological grading is not.

The analysis of survival times shows similar results: all the morphological mean features, except roundness, have significant association with survival (Table III). Note that for each feature, mean values greater than the 66.7 percentile of their distribution are associated with worst prognosis; the hazard ratios compared with values below the 33.3 percentile, range from 1.64 for mean nuclear longest axis to 3.752 for VPE. In particular strong associations are seen with mean nuclear perimeter, mean nuclear area, VPE and mitotic index.

Very strong associations are also seen for FIGO stage, response to chemotherapy and mass of residual disease. FIGO stage IV appears to have a hazard ratio compared with stage III of 3.988; in fact none of FIGO stage IV patients survived the 5 year post-operative period and the median survival period was only 19 months. Similarly, progressive disease is, as expected, a bad prognostic characteristic having a hazard ratio compared with complete remission of 3.654; 4 (14.8%) of the 27 patients with progressive disease survived the 5 year post-operative period (compared with 54.5% for patients with complete remission) and their median survival period was 23 months. The size of residual disease is also a bad prognostic factor, having a hazard ratio, compared with non-residual disease, of 2.869, survival percentage 17.9 (compared with 53.1% for non-residual disease) and median survival period 24.5 months.

### *Multivariate analysis*

*Survival* vs *non-survival* Discriminant analysis and logistic regression produced the same best combination of prognostic features: mean nuclear area, mean VPE, s.d. of VPE, FIGO stage and mean nuclear perimeter. The resulting linear function (discriminant or regression respectively) or in this case the optimum prognostic score (OPS) was as follows:

$$\text{OPS} = -16.999 + (1.361 \times \text{mean VPE}) + (19.964 \times \text{s.d. of VPE}) + (0.026 \times \text{mean nuclear area}) + (18.897 \times \text{FIGO (coded as 0 if FIGO is III and 1 if FIGO is IV)}) (-0.736 \times \text{mean nuclear perimeter})$$

On the basis of this score, one can classify an observation in the survivors group if OPS $<0$ and in the non-survivors if OPS $>0$. With our sample reclassification results were as shown in Table IV. The overall proportion of correct reclassification of observations was 90.0%.

The Kaplan–Meier estimates of survival curves for the two groups (OPS $<0.0$ and OPS $>0.0$) are shown in Figure 8. Their Mantel–Cox statistic was highly significant ($P<0.00001$); the median for the group with bad prognosis (OPS $>0.0$) was 24 months.

*Survival analysis* Cox's proportional hazards model fitted stepwise pointed out the following best combination of prognostic features: FIGO stage, mitotic index, mean VPE, s.d. of longest nuclear axis, s.d. of VPE and s.d. of nuclear area. The regression coefficients, standard errors, $P$-values and corresponding hazard ratios are shown in Table V. Concerning response to chemotherapy, the morphometric features that were significantly different between responders and non-responders are shown in Tables VI and VII.

Multivariate analysis (discriminant analysis and logistic regression) of our data revealed the following best combination of prognostic features: mean nuclear area, mean nuclear longest axis and mean nuclear shortest axis. The resulting optimum prognostic score is as follows:

$$\text{OPS} = 292.9 + (0.076 \times \text{mean nuclear area}) - (4.722 \times \text{mean nuclear longest axis}) - (3.789 \times \text{mean nuclear shortest axis})$$

when the patients with positive value of OPS are expected to benefit from a cisplatin combination chemotherapy in contrast to those with negative OPS, who will not.

### Discussion

The 5 year survival rate for ovarian carcinomas depends on the stage of the disease: 70% for stage I, 25% for stage II, 12% for stage III, 0% for stage IV (Friedlander *et al.*, 1991). Adjuvant chemotherapy is therefore indicated especially for

**Table IV**

| *Actual group* | *Optimum prognostic score* $<0.0$ | $>0.0$ |
|---|---|---|
| Survivors ($n = 22$) | 22 (100.0%) | 0 (0.0%) |
| Non-survivors ($n = 38$) | 6 (15.8%) | 32 (84.2%) |

**Table V**

| Variable | Coefficient | s.e. | P | Hazard ratio |
|---|---|---|---|---|
| FIGO stage (0 for III and 1 for IV) | 2.110 | 0.414 | <0.001 | 8.247 |
| Mitotic index | 0.022 | 0.008 | 0.008 | 1.022 |
| Mean VPE | 0.077 | 0.017 | <0.001 | 1.080 |
| s.d. of nuclear longest axis | – 16.690 | 5.280 | 0.002 | 0.057 |
| s.d. of VPE | 0.897 | 0.333 | 0.007 | 2.452 |
| s.d. of nuclear area | 4.560 | 2.190 | 0.037 | 95.570 |

**Table VI** Differences in morphometric features between responders and non-responders to chemotherapy

| Feature | n | Mean | Median | $P^a$ | $P^b$ |
|---|---|---|---|---|---|
| Mean nuclear longest axis | | | | | |
| Responders | 33 | 88.43 | 87.15 | 0.0341 | 0.249 |
| Non-responders | 27 | 91.07 | 93.28 | | |
| Mean nuclear shortest axis | | | | | |
| Responders | 33 | 80.44 | 78.82 | 0.034 | 0.012 |
| Non-responders | 27 | 86.40 | 87.30 | | |
| Mean nuclear perimeter | | | | | |
| Responders | 33 | 281.53 | 278.43 | 0.049 | 0.028 |
| Non-responders | 27 | 298.36 | 303.29 | | |
| Mean nuclear area | | | | | |
| Responders | 33 | 5325.46 | 5349.56 | 0.001 | 0.002 |
| Non-responders | 27 | 6361.45 | 6453.48 | | |
| Mean roundness | | | | | |
| Responders | 33 | 0.9010 | 0.907 | 0.136 | 0.141 |
| Non-responders | 27 | 0.8916 | 0.894 | | |
| Mean VPE | | | | | |
| Responders | 33 | 32.708 | 30.096 | 0.489 | 0.323 |
| Non-responders | 27 | 30.120 | 23.939 | | |
| Mitotic index | | | | | |
| Responders | 33 | 22.234 | 18.500 | 0.128 | 0.143 |
| Non-responders | 27 | 29.738 | 25.810 | | |

[a]*t*-test; [b]Wilcoxon's rank-sum test.

**Table VII** Single clinical and morphometric features and their independent prognostic value concerning response to chemotherapy; only features with significant independent prognostic value are shown

| Feature | n | Responded (%) | P |
|---|---|---|---|
| Residual disease | | | |
| <2 cm | 32 | 23 (71.9%) | 0.01 |
| ⩾2 cm | 28 | 10 (35.7%) | |
| Mean nuclear area | | | |
| – 5129.8 | 20 | 15 (75.0%) | 0.0006 |
| 5129.8 – 6393.9 | 20 | 14 (70.0%) | |
| 6393.9 + | 20 | 4 (20.0%) | |
| s.d. of nuclear area | | | |
| – 1.241 | 20 | 17 (85.0%) | 0.0019 |
| 1.241 – 1.338 | 20 | 10 (50.0%) | |
| 1.338 + | 20 | 6 (30.0%) | |

advanced epithelial ovarian cancer in addition to surgery. Recent studies suggest that cisplatin treatment improves prognosis in approximately 30% of FIGO III and IV epithelial ovarian cancer patients (Perez *et al.*, 1993). However, the outcome of treatment is not only determined by the treatment itself but also by other parameters such as clinical and morphometric observations. It is important therefore to recognise these factors in order to identify patients at high risk who may require aggressive treatment in order to improve their survival.

Morphometry of ovarian carcinomas was studied mainly by two groups of investigators (Baak *et al.*, 1981, 1985, 1986*a*, 1986*b*, 1988; Haapasalo *et al.*, 1989, 1991). In 1988 Baak *et al.* evaluated in 73 ovarian cancers the prognostic significance of morphometric features and DNA content in comparison with histological type, grade of differentiation and a number of clinical characteristics. They concluded that 'nuclear size is an important predictor of the sensitivity of tumour cells to cisplatin treatment' although 'it is not quite clear which underlying cell-biological mechanism it reflects'.

In 1989 Haapsalo *et al.* estimated the morphometric parameters in 105 ovarian carcinomas. Morphometric parameters included mitotic activity index, volume corrected mitotic index (M/V), volume fraction of neoplastic epithelium, nuclear area, nuclear perimeter, shortest and longest nuclear axis and form factor of nucleus. Their results indicated that clinical stage was the best predictor of prognosis followed by the M/V index. The latter was the best prognostic factor in all the tumour subgroups studied. Regarding VPE their results were different from the earlier paper of Baak *et al.* (1986). They indicate as a possible reason that in Baak's material about one-third of the carcinomas were mucinous whereas in their material only four of the cases were mucinous carcinomas.

In our study FIGO III and IV epithelial ovarian cancer uniformly treated patients have been included and our results fulfil the demand of an accurate prognostic test based on clinical and reproducible quantitative pathological features. Our material can readily be compared with the incidence of the histological tumour types mentioned in the literature (DiSaia *et al.*, 1993).

According to our results patients with low values of VPE or mitotic index seem to have a good prognosis concerning survival or not at 5 years (Figures 6 and 7). These findings are in accordance with those reported in the literature (Baak *et al.*, 1988; Haapasalo *et al.*, 1989). Regarding treatment with cisplatin, these features were found not quite significant when used for the identification of patients treated with cisplatin. Many of these patients survived even if they did not respond to the regimen used perhaps because of different cell biological mechanisms.

Another factor of great importance is the size of the residual tumour (Figure 2). The prognosis, as is generally accepted, was found to be favourable if the diameter of the largest residual mass did not exceed 2 cm and/or if there were fewer than 20 sites of disease, regardless of the bulkiness of the disease.

Our results also indicate that nuclear size (Figures 3–5) is an important predictor of the response of tumour to cisplatin chemotherapy. In this aspect the results are in agreement with those of Baak *et al.* (1988), although the regimen and dosage of treatment of our patients is different. Many authors have compared the two regimens (cisplatin, cyclophosphamide and doxorubicin used by Baak's group and cisplatin, cyclophosphamide). Some of them were unable to demonstrate any difference in overall response rate, in rate of pathological response and in survival (Edmonson *et al.*, 1985; Neijt *et al.*, 1987; Omura *et al.*, 1989). Others reported higher rate of complete response or improved survival using cisplatin, cyclophosphamide and doxorubicin (Jakobsen *et al.*, 1985; Bruzzone *et al.*, 1990). It is difficult to compare the results of these studies because of the different dose intensities used. It is clear that the combination of cisplatin with cylcophosphamide may produce as high a response rate as combinations with other drugs (doxorubicin, hexamethylmelamine) without their potential cardiac or neurological side-effects.

It appears therefore that nuclear dimension is a significant predictor regardless of the dosage or the regimen used, provided that cisplatin is included.

**References**

BAAK JPA, AGRAFOJO BLANCO A AND KURVER PHJ. (1981). Quantitation of borderline and malignant mucinous ovarian tumours. *Histopathology*, **5**, 353-360.

BAAK JPA, FOX J, LANGLEY FA AND BUCKLEY H. (1985). The prognostic value of morphometry in ovarian epithelial tumours of borderline malignancy. *Int. J. Gynecol. Path.*, **4**, 186-191.

BAAK JPA, WISSE-BREKELMANS ECM AND LANGLEY FA. (1986*a*). Morphometric data to FIGO stage and histological type and grade for prognosis of ovarian tumours. *J. Clin. Pathol.*, **39**, 1340-1346.

BAAK JPA, LANGLEY FA AND TALERMAN A. (1986*b*). Interpathology and intrapologist disagreement in ovarian tumour grading and typing. *Anal. Quant. Cytol. Histol.*, **8**, 354-357.

BAAK JPA, STOLK JG, CHAN KK AND KENEMANS P. (1987). Prognostic factors in borderline and invasive ovarian tumours of the common epithelial type. *Pathol. Res. Pract.*, **182**, 755-774.

BAAK JPA, SCHIPPER NW, WISSE-BREKELMANS ECM, CEELEN TH, BOSMAN FT, VAN GEUNS H AND WILS J. (1988). The prognostic value of morphometrical features and cellular DNA content in cisplatin-treated late ovarian cancer patients. *Br. J. Cancer*, **57**, 503-508.

BAAK JPA. (1991). Morphometric and combined morphometric-DNA cytometric applications-the female reproductive tract, Ovary. In *Manual of Quantitative Pathology in Cancer Diagnosis and Prognosis*. Baak JPA (ed.) pp. 345-352. Springer-Verlag: Heidelberg.

BAAK JPA, WALBOOMERS JMM AND OUDEJANS CBM. (1992). Quantitative pathology. In *Gynecologic Oncology*, Malcolm Coppleson (ed.) pp 107-118. Churchill Livingstone: Edinburgh.

BRUZZONE M, REPETTO L, CHIARA C, OLIVA C, GARDIN G, CONTE PF AND ROSSO R. (1990). A randomised trial comparing PC vs PAC chemotherapy in epithelial ovarian cancer: 7 years follow-up (Abstract). *Proc. Am. Soc. Clin. Oncol.*, **9**, 157.

COX DR. (1970). *Analysis of Binary Data*. Chapman and Hall: London.

COX DR. (1972). Regression models and life - tables. *J. R. Stat. Soc. Series B*, **34**, 187-202.

DISAIA PJ AND CREASMAN WT. (1993). Epithelial ovarian cancer. In *Clinical Gynecologic Oncology*, Manning S (ed.) pp. 333-425. Mosby Year Book: St Louis, MO.

EDMONSON JH, MCCORMICK GW, FLEMING TR, CULLINAN SA, KROOK JE, MALKASIAN GD, PODRATZ KC, MAILLIARD JA, JEFFRIES JA AND BARLOW JF. (1985). Comparison of cyclophosphamide plus cisplatin versus hexamethylmelamine, cyclophosphamide and cisplatin in combination as initial chemotherapy for stage III and IV ovarian carcinomas. *Cancer Treat. Rep.*, **69**, 1243-1248.

EGRET Version 0.26.06 (1993). Statistics and Epidemiology Research Corporation: Seattle, WA.

FLEEGE JC, VAN DIEST PJ AND BAAK JPA. (1991). Reliability of quantitative pathological assessments, standards and quality control. In *Manual of Quantitative Pathology in Cancer Diagnosis and Prognosis*. Baak JPA (ed.) pp. 151-181. Springer-Verlag: Heidelberg.

FRIEDLANDER ML AND DEMBO AJ. (1991). Prognostic factors in ovarian cancer. In *Seminars in Oncology*. Yarbro JW (ed.) pp. 205-212. WB Saunders: Philadelphia.

HAAPASALO H, COLLAN Y, ATKIN NB, PESONEN E AND SEPPA A. (1989). Prognosis of ovarian carcinomas: prediction by histoquantitative methods. *Histopathology*, **15**, 167-178.

HAAPASALO H, ATKIN NB, COLLAN Y, PESONEN E AND PALJARVI L. (1991). Tumour ploidy, morphometry, histological grading and clinical features in ovarian carcinoma: mutual relations. *Anal. Cell Pathol.*, **3**(5), 261-271.

JAKOBSEN A, BERTELSEN K, SELL A, STROVER I AND PETERSEN M. (1985). Advantage of CAP over CP in terms of survival in advanced ovarian carcinoma (Abstract). *Proc. Am. Soc. Clin. Oncol.*, **4**, 113.

KALBFLEISCH JD AND PRENTICE RL. (1980). *The Statistical Analysis of Failure Time Data*, Wiley: London.

KAPLAN EL AND MEIER P. (1958). Nonparametric estimation from incomplete observations. *J Am. Stat. Assoc.*, **53**, 457-481.

MORISSON DF. (1976). *Multivariate Statistical Methods*. 2nd edn, McGraw-Hill.

NEIJT JP, TEN BOKKEL HUININK WW, VAN DER BURG MEL, VAN OOSTEROM AT, WILLEMSE PH, HEINTZ AP, VAN LENT M, TRIMBOS JB, BOUMA J AND VERMORKEN JB. (1987). Randomised trial comparing two combination chemotherapy regimens (CHAP-S vs CP) in advanced ovarian carcinoma. *J. Clin. Oncol.*, **5**, 1157-1168.

OMURA GA, BUNDY BN, BEREK JS, SMITH JF AND HEINTZ A. (1989). Randomised trial of cylcophosphamide plus cisplatin with or without doxorubicin in ovarian carcinoma: A Gynecologic Oncology Group study. *J. Clin. Oncol.*, **7**, 457-465.

PEREZ RP, HAMILTON TC, OZOLS RF AND, YOUNG RC. (1993). Mechanisms and Modulation of Resistance to chemotherapy in ovarian cancer. *Cancer Suppl.* **71**, 1571-1580.

ROLLANSON TP. (1992). Prognostic factors in ovarian cancer. In *Recent Advances in Histopathology*, Anthony PP, MacSween RNH (eds). pp. 200-201. Longman: England.

SPSS Release 5.4 (1992). SPSS. Chicago.

VLACHONIKOLIS IG AND MARRIOTT FHC. (1982). Discrimination with mixed binary and continuous data. *Appl. Stat.*, **31**, 23-31.